# Temporal Characterization of Blood–Brain Barrier Disruption with High-Frequency Electroporation

**DOI:** 10.3390/cancers11121850

**Published:** 2019-11-23

**Authors:** Melvin F. Lorenzo, Sean C. Thomas, Yukitaka Kani, Jonathan Hinckley, Matthew Lee, Joy Adler, Scott S. Verbridge, Fang-Chi Hsu, John L. Robertson, Rafael V. Davalos, John H. Rossmeisl

**Affiliations:** 1 Bioelectromechanical Systems Laboratory, School of Biomedical Engineering and Sciences, Virginia Tech-Wake Forest University, Blacksburg, VA 24061, USA; mflorenz@vt.edu (M.F.L.); lmatt9@vt.edu (M.L.); davalos@vt.edu (R.V.D.); 2 Department of Biomedical Engineering and Mechanics, Virginia Tech, Blacksburg, VA 24061, USA; seanct@vt.edu (S.C.T.); sverb@vt.edu (S.S.V.); drbob@vt.edu (J.L.R.); 3 Department of Small Animal Clinical Sciences, Virginia Tech, Blacksburg, VA 24061, USA; yukitaka@vt.edu (Y.K.); hinckley@vt.edu (J.H.); joya10@vt.edu (J.A.); 4 Department of Biostatistics and Data Science, Division of Public Health Sciences, Wake Forest School of Medicine, Winston-Salem, NC 27157, USA; fhsu@wakehealth.edu

**Keywords:** blood–brain barrier disruption, electric field threshold, Evans blue dye, high-frequency electroporation, focal therapy, gadopentetate dimeglumine, electropermeabilization, numerical modeling, BBB disruption temporal threshold, transient BBB disruption

## Abstract

Treatment of intracranial disorders suffers from the inability to accumulate therapeutic drug concentrations due to protection from the blood–brain barrier (BBB). Electroporation-based therapies have demonstrated the capability of permeating the BBB, but knowledge of the longevity of BBB disruption (BBBD) is limited. In this study, we quantify the temporal, high-frequency electroporation (HFE)-mediated BBBD in an in vivo healthy rat brain model. 40 male Fisher rats underwent HFE treatment; two blunt tipped monopolar electrodes were advanced into the brain and 200 bursts of HFE were delivered at a voltage-to-distance ratio of 600 V/cm. BBBD was verified with contrast enhanced T1W MRI (gadopentetate dimeglumine) and pathologically (Evans blue dye) at time points of 1, 24, 48, 72, and 96 h after HFE. Contrast enhanced T1W scans demonstrated BBBD for 1 to 72 h after HFE but intact BBB at 96 h. Histologically, tissue damage was restricted to electrode insertion tracks. BBBD was induced with minimal muscle contractions and minimal cell death attributed to HFE. Numerical modeling indicated that brief BBBD was induced with low magnitude electric fields, and BBBD duration increased with field strength. These data suggest the spatiotemporal characteristics of HFE-mediated BBBD may be modulated with the locally applied electric field.

## 1. Introduction

The blood–brain barrier (BBB) is an active and highly selective biological barrier made up by the complex interactions between brain capillary endothelial cells (BCECs), astrocytes, tight junction (TJ) proteins, and other supportive cells; together these components regulate molecular transport across the microvasculature in the central nervous system (CNS) [1,2]. Coupled with molecular efflux transporters (such as P-glycoproteins and multidrug resistance protein 1) expressed on the surface of specialized BCECs, the intact BBB acts to maintain brain homeostasis and isolates the CNS from circulating pathogens. Though effective in this regard, the BBB also hinders transport of therapeutic drugs and large molecules, thereby impeding treatment of intracranial malignancies [3,4]. Treatment options for Parkinson’s disease, Alzheimer’s disease, and brain tumors are limited due to the inability to accumulate drug concentrations required to achieve a clinically relevant response. Therefore, the ability to induce predictable, prolonged BBB disruption (BBBD) is critical to the advancement of modern therapeutics in the treatment of intracranial malignancies.

One potential solution for intracranial drug delivery is convection-enhanced delivery (CED), which seeks to bypass the BBB by direct therapeutic administration to the brain parenchyma and target tissue. CED uses pressure-driven flow through an array of catheters to deliver a variety of potential agents, including nanoparticles (~200 nm) [5], small molecules (<1 kDa) [6], and monoclonal antibodies [7], to large volumes of brain parenchyma [8]. While effective, CED is limited by the occurrence of perfusate reflux and by the requirement for lengthy treatment sessions due to relatively slow infusion rates.

Another approach, transcranial focused ultrasound (FUS) combined with microbubbles, has been shown to disrupt the BBB through disassembly of TJ proteins occludin, claudin-5, and ZO-1 [9]. While FUS offers versatile and non-invasive BBBD, BBB recovery typically occurs within a few hours after FUS [10], potentially limiting practical treatment windows. These drawbacks highlight the need for alternative approaches for enhanced parenchymal drug delivery.

Irreversible electroporation (IRE) is a well-established focal ablation therapy for pancreatic cancer, hepatocellular carcinoma, and other cancers [11,12,13,14,15,16]. IRE offers an attractive alternative to thermal therapies, as cells are ablated with high intensity (up to 3000 V), short duration (50–100 µs) pulsed electric fields (EFs). It has been repeatedly observed that if tissues are exposed to EFs above a critical value, cell death with high demarcation and minimal-to-no thermal necrosis is achieved [17]. Since IRE is an EF-dependent ablation therapy, modification of pulsing parameters and the electrode configuration allows for clinically relevant tissue ablation (>3 cm^3^) with minimal Joule heating effects [18,19,20] and preservation of tissue infrastructure such as blood vessels and ducts [21,22]. Preclinical studies in a spontaneous canine brain tumor model demonstrated safe, reproducible, and clinically relevant ablations with minimal side effects attributed to IRE therapy [23,24,25].

In addition to the development of a nonthermal lesion, IRE induces voltage-dependent secondary volumes of BBBD in cerebral tissues [26,27,28], demonstrating the potential for using IRE as an effective intracranial combinatorial therapy to facilitate drug diffusion into the brain parenchyma. Monophasic IRE using 90 50 µs pulses at 1 Hz with applied voltage-to-distance (V/d) ratios of 200 and 400 V/cm has succeeded in disrupting the BBB with limited detriment to the viability of the surrounding tissue [26]. Using these parameters, BBBD was achieved at an electric field threshold (EFT) of 330 V/cm, notably lower than the 500 V/cm EFT for IRE [28]; this delineation points to the possibility of BBBD for targeted drug delivery without the need for lethal quantities of energy application. More recently, the EFTs for BBBD and IRE ablation were quantified as a function of pulse number; it was demonstrated that an increase in the number of pulses decreased the EFT for BBBD [29]. This effect is mirrored in vitro, where a monolayer of endothelial cells was transiently permeabilized but remained viable [30]. Interestingly, even application of low-voltage monophasic pulses, with no detectable electroporation of the cell monolayer, leads to an increase in paracellular passage of small molecules [31].

Pre-clinical studies in our laboratories have found IRE to be effective at both BBB permeabilization and tissue ablation, but patients required intraoperative neuroparalytics and general anesthesia to reduce unintended muscle contractions. This could limit the clinical practice of otherwise applicable surgical approaches, such as intraoperative brain mapping for IRE tissue ablation, that require the absence of general anesthesia. To improve the versatility of IRE for therapeutic applications, our group has developed a novel tumor ablation strategy which utilizes bursts of bipolar pulsed EFs (0.5–10 µs) to nonthermally ablate tumors [32,33]. This second-generation strategy, termed high-frequency IRE (H-FIRE), reduces the potential for nerve and muscle excitation by applying alternating polarity pulses, obviating in many cases the need for paralytics and cardiac synchronization [34,35,36]. Prior to the onset of electroporation, the high frequency waveforms of H-FIRE penetrate to the intracellular domain more efficiently than their low frequency counterparts used in IRE; this phenomenon has been exploited to target cells with enlarged nuclei, a physical hallmark common in malignant cells, and resulted in lower lethal EFTs in tumor cells [37,38]. More promising still, these higher frequency waveforms mitigate electrical heterogeneities in complex tissues, resulting in more predictable ablations in highly heterogenous tissues such as tumor tissue [39,40,41,42].

Our group has observed that H-FIRE, like traditional IRE, induces focal tissue ablation with a surrounding zone of BBBD that extends centimeters beyond the nonthermal lesion [35,43]. Thus, in the interest of future H-FIRE human clinical trials, in which H-FIRE-mediated BBBD will target the peritumoral penumbra of normal appearing brain, we focus our study to quantify the effects of high-frequency electroporation (HFE, to distinguish it from H-FIRE, an ablative technology) on BBBD in an in vivo healthy rodent model. Specifically, we seek to elucidate the duration of HFE-mediated BBBD in vivo. By utilizing a two-needle configuration, the applied EF is non-uniform and generates a gradient of local EFs across the tissue, enabling investigation of BBBD by electric fields of varied strength. Prior work has revealed paracellular BBBD with low pulsed EFs [31]; transcellular BBBD with reversible electroporation [30]; and tissue ablation, which affects both transcellular and paracellular passage, with irreversible electroporation [23]. We hypothesize that the duration of BBBD with HFE is dependent on the local EF, likely leading to BBBD through various mechanisms (i.e., TJ disruption, cell electroporation, cell irreversible electroporation). The data presented here support HFE as a unique tool for permeating the BBB, with control over the spatiotemporal characteristics through modifications of the local EFs.

## 2. Results

HFE treatment was administered across two blunt tipped monopolar electrodes using a custom bipolar pulse generator at a V/d ratio of 600 V/cm, energized time 100 µs, burst scheme 5-5-5 µs, and 200 bursts. BBB permeability was assessed by intraperitoneal injection of a solution formulated with gadopentetate dimeglumine and Evans blue dye (Gd-EBD); this solution was administered 1 hour prior to sacrifice to allow for circulation and clearance of the Gd-EBD solution. Gd and EBD are too large to permeate the intact BBB; their presence in the brain parenchyma would indicate BBBD. The temporal BBBD characterization included time points 1, 24, 48, 72, and 96 h post-HFE. Quantification of EBD fluorescence in intraparenchymal tissue and blood serum further characterized HFE-mediated BBBD. In all cases, the values reported are in the format mean ± standard deviation.

### 2.1. High-Frequency Electroporation BBBD Analysis

The sham-operated group showed no Gd-EBD uptake. There was no visible EBD staining present in the tissue sections. No Gd uptake within the brain parenchyma in post-contrast T1W MRI scans was detected. Quantification of the sham serum EBD fluorescence (1494 ± 25.5 µg/g, *n* = 1) and intracranial EBD fluorescence (0.2 ± 0.03 µg/g, *n* = 1) further indicated minimal EBD uptake into the sham brain parenchyma (Table 1). Tissue damage was restricted to the electrode insertion tracks when H&E-stained sections were examined (Figure 3).

#### 2.1.1. Temporal BBBD Characterization

**Figure 1:**
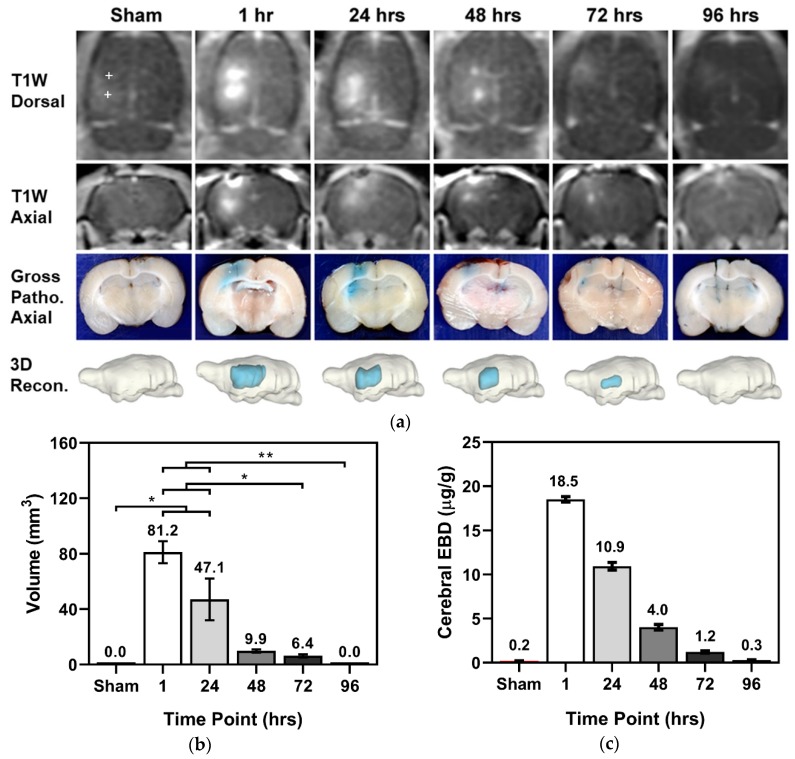
Visualization of long-lived BBBD resulting in significant diffusion of normally impermeant Gd-EBD. 200 bursts of HFE were applied across two monopolar electrodes with 4 mm spacing; each burst was energized for 100 µs, and a V/d ratio of 600 V/cm was applied. Gd-EBD was administered systemically and allowed to circulate for 1 hour prior to sacrifice. (**a**) Depiction of BBBD, as seen in contrast enhanced T1W MRI scans, tissue sections with EBD staining, and subsequent 3D reconstruction, in the sham and at timepoints 1 hour, 24 h, 48 h, 72 h, and 96 h post-HFE treatment; a “+” symbol in the T1W Dorsal view denotes the electrode insertion track for the sham. Without HFE, no uptake of Gd-EBD is seen. All images depict representative scans/tissue sections of BBBD either along the electrode insertion track or in a plane orthogonal to the electrode tip. (**b**) Volumetric measurements determined from tissue sections and (**c**) quantification of intracranial EBD fluorescence show an exponential decrease in BBBD following HFE. * denotes a *p*-value < 0.05 and ** denotes a *p*-value < 0.01.

HFE for treatment groups 1–4 demonstrated BBB permeability to Gd-EBD (Figure 1a). In Figure 1, a “+” symbol in the T1W Dorsal view denotes the electrode insertion tracks for the sham; the trajectory of electrode insertion was consistent in all groups. All images in Figure 1 depict representative scans/tissue sections of BBBD either along the electrode insertion track or in a plane orthogonal to the electrode tip. Maximal BBBD volume was achieved at 1 h (81.2 ± 7.9 mm^3^, *n* = 3) post-HFE, followed by an exponential decrease at 24 (47.1 ± 15.1 mm^3^, *n* = 7), 48 (9.9 ± 1.1 mm^3^, *n* = 8), 72 (6.4 ± 1.1 mm^3^, *n* = 8), and 96 h (0.0 ± 0.0 mm^3^, *n* = 4), as measured in gross pathological tissue sections (Figure 1b). A Kruskal–Wallis (KW) test demonstrated that the mean pathological BBBD volume in at least one group is different from the others (*p* < 0.0001) within the temporal arm of this study. A post hoc Dunn’s demonstrated that the 1 h (p = 0.019) and 24 h (*p* = 0.0401) groups had BBBD volumes significantly different than that of the sham group. It should be noted that in the 1 h and 24 h timepoints, contrast was inadvertently injected into the intestines of 1 rat per treatment group, reducing to sample size of each group to *n* = 3 and *n* = 7, respectively. In T1W MRI, volumetric measurements were as follows: 0.0 ± 0.0 mm^3^ in the sham group (*n* = 2), 84.1 ± 8.7 mm^3^ at 1 h (*n* = 2), 40.9 ± 5.4 mm^3^ at 24 h (*n* = 4), 10.4 ± 1.1 mm^3^ at 48 h (*n* = 4), 5.8 ± 1.0 mm^3^ at 72 h (*n* = 4), and 0.0 ± 0.0 mm^3^ at 96 h (*n* = 2). A Kruskal–Wallis (KW) test demonstrated that the mean MRI-derived BBBD volume in at least one group is different from the others (*p* = 0.0083) within the temporal arm of this study. A post hoc Dunn’s demonstrated that the 1 h (*p* = 0.0261) group had a BBBD volume significantly different than that of the sham group. Notably, there is a strong correlation between the BBBD volumes measured in both post-contrast T1W MRI scans and tissue sections; the paired-t test demonstrated no statistical differences (*p* = 0.8357) between volumetric analysis methods within treatment groups (Table 1).

In addition to the volumetric characterization of BBBD, the fluorescence of EBD within the brain parenchyma and within blood serum was quantified. In all groups, including the sham, the serum EBD fluorescence was at least 1318.3 ± 61.6 µg/g; KW indicated no significant differences in the serum EBD for all treatment groups (*p* = 0.886) for 1 (*n* = 2), 24 (*n* = 4), 48 (*n* = 4), 72 (*n* = 4), and 96 h (*n* = 2). While there was ample systemic EBD, measurements of parenchymal EBD fluorescence in the sham group (0.2 ± 0.03 µg/g) indicated minimal uptake following electrode insertion. Intraparenchymal EBD indicated a maximum fluorescence measured at 1 hour (18.5 ± 0.30 µg/g, *n* = 2), followed by an exponential decay at 24 (10.9 ± 0.44 µg/g, *n* = 4), 48 (4.0 ± 0.31 µg/g, *n* = 4), 72 (1.2 ± 0.13 µg/g, *n* = 4), and 96 h (0.3 ± 0.05 µg/g, *n* = 2) (Figure 1c). It should be noted only a single sham EBD fluorescence measurement was recorded; the KW test indicated the mean EBD fluorescence of at least one group was different from that of the rest (*p* = 0.0089), but a post hoc Dunn’s test failed to reveal statistical difference between any particular set of groups. This is likely due to a sham sample size of *n* = 1 for EBD fluorescence measurements.

#### 2.1.2. BBBD with Varied V/d and Burst Number

In addition to the temporal study, the effect of applied V/d ratio was investigated at the 1 h timepoint. Pathological analysis of BBBD volume due to V/d ratios 0 (sham), 600 (Group 1), and 1200 V/cm (Group 6, H-FIRE protocol, *n* = 4) indicated at least one group experienced a different BBBD volume (KW test *p* = 0.0048). A V/d ratio of 1200 V/cm (Group 6) resulted in a significant increase in Gd-EBD uptake compared to the sham group (*p* = 0.0332, Dunn’s test), with the H-FIRE protocol having a BBBD volume of 113.8 ± 7.1 mm^3^, as measured and evaluated in tissue sections. Quantification of intracranial EBD fluorescence demonstrated more EBD within the brain parenchyma of the 1200 V/cm group (25.1 ± 1.27 µg/g, *n* = 2) than in the 600 V/cm group; however, a KW test determined no statistically significant difference (*p* = 0.200), likely due to a sham sample size *n* = 1 for EBD fluorescence.

We also compared the effects of burst number 0 (sham), 200 (Group 1), and 100 (Group 7, *n* = 4) on BBBD volume; a KW test to this effect yielded *p* = 0.0429, meaning at least one group had a true mean different from another. A decrease in number of bursts from 200 (Group 1) to 100 (Group 7) had minimal impact (*p* > 0.9999, Dunn’s test) on Gd-EBD uptake, with the latter having a BBBD volume 87.0 ± 5.6 mm^3^, as measured and evaluated in tissue sections. Quantification of intracranial EBD fluorescence did not vary significantly between the 100-burst group (18.8 ± 0.88 µg/g, *n* = 2) and the 200-burst group; a KW test determined no statistical differences (*p* = 0.600), likely due to a sham sample size *n* = 1 for EBD fluorescence.

### 2.2. Numerical BBBD Analysis

**Figure 2:**
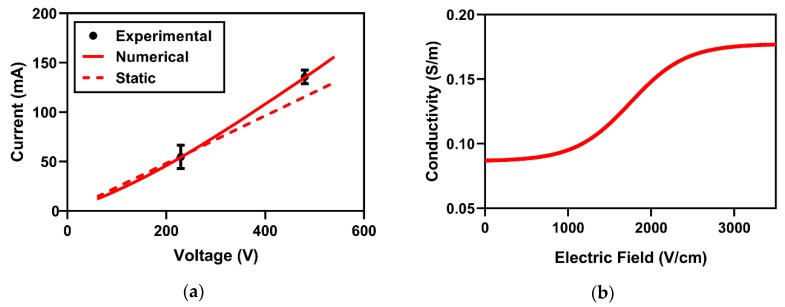

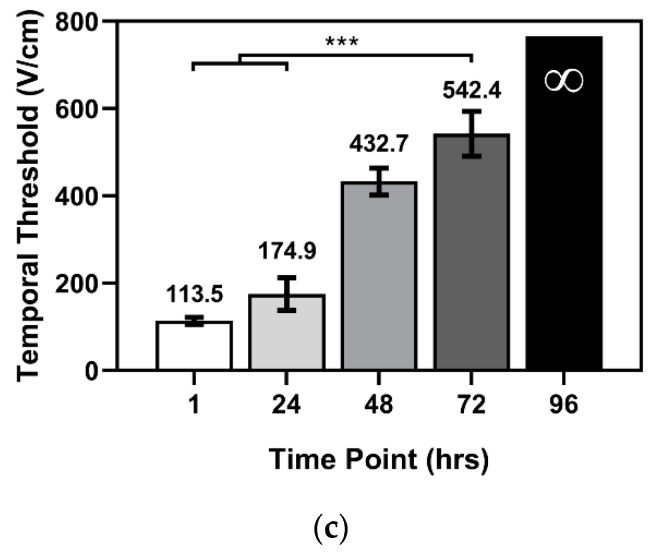
Numerical validation and BBBD temporal thresholds. Numerical validation was accomplished by comparing the (**a**) electric currents from experimental and numerical approaches. This analysis resulted in a (**b**) sigmoidal electrical conductivity response from HFE treatment. BBBD temporal thresholds were determined as the electric field contours enclosing a volume equivalent to the measured BBBD volumes from tissue sections for (**c**) timepoints 1 h, 24 h, 48 h, 72 h, and 96 h after HFE treatment. *** denotes a *p*-value < 0.001.

A numerical model was constructed to approximate the electric field distribution during HFE-treatment; to accomplish this, the changes in electrical conductivity with varied local EF needed to be determined. This conductivity relationship was established by matching the first-burst experimental and numerical electric currents (Figure 2a). The sigmoidal conductivity response which best fits Equation (2) corresponds to conductivity values of σ_0_ = 0.087 S/m and σ_f_ = 0.178 S/m (Figure 2b). This sigmoid was implemented across the 3D reconstructed rat brain domain, and the resulting EF distribution is depicted in Figure 6e.

Following the approximation of the electric field distribution, numerical methods were implemented to quantify electric fields corresponding to permeated BBB volumes at various timepoints; these thresholds will be referred to throughout the text as the BBBD temporal thresholds (BTTs). The analysis to determine BTTs assumes the measured BBBD volumes are topologically consistent with computed EF contour. Therefore, a volume integration was implemented to determine the volumes of tissues exposed to EFs between 25 V/cm and 1000 V/cm in increments of 0.1 V/cm. These values were then matched to the experimental BBBD volumes from tissue section measurements; the closest matching values are reported as the BTTs (Figure 2c). The 1 hour timepoint corresponded to the lowest threshold (113.5 ± 8.2 V/cm, *n* = 3), followed by higher BTTs in the 24 (174.9 ± 37.1 V/cm, *n* = 7), 48 (432.7 ± 30.8 V/cm, *n* = 8), and 72 (542.5 ± 51.5 V/cm, *n* = 8) h timepoints. In the sham group and the 96 h timepoint group, no BBBD was seen. A KW test (*p* < 0.001) followed by a post hoc Dunn’s test demonstrated a statistically significant difference between the BTTs at 1 h and 72 h (*p* = 0.0005) and at 24 h and 72 h (*p* = 0.0006).

### 2.3. Histomorphologic Analyses

**Figure 3:**
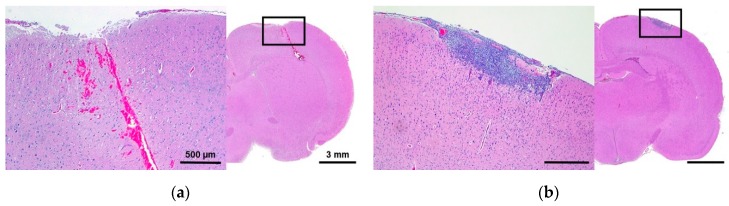

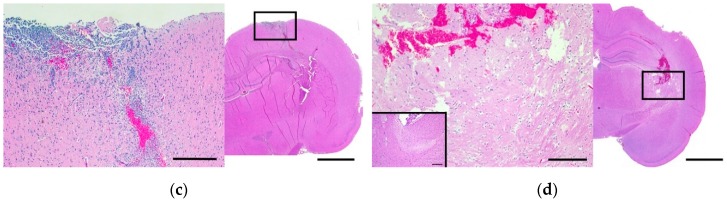
Representative H&E staining of histologic grades observed in study. Following the grading criteria outlined in Table 4, the following images depict a score of (**a**) Grade 1, (**b**) Grade 2a, (**c**) Grade 2b, and (**d**) Grade 4 compared to untreated contralateral brain shown as an inset in the bottom left corner of 5d. The scales 500 µm and 3 mm are consistent across each image.

Histologic changes in the brain were graded using a modified grading scheme to quantify tissue damage; this scheme is outlined in the Methods section below. In this scheme, grade 1 scoring indicates superficial tissue damage restricted to the electrode insertion tracks, whereas grade 4 scoring indicates regional parenchymal tissue necrosis remote from the electrode insertion tracks. The lesions observed in the histological slides were consistent within each treatment group, and a summary of these results is given in Table 2. All treatment groups demonstrated superficial lesions due to the mechanical pressure applied during electrode insertion, as demonstrated in Figure 3. Grade 1 scoring (Figure 3a) was observed in the sham-operated group; in the Burst_100_ group (Group 7); and in the immediate response group, at timepoints 1, 24, and 48 h. Grade 2 scoring was observed in the delayed response group (72 and 96 h), though there was a varying degree of meningeal inflammation (Figure 3b,c). Grade 3 and 4 scoring was observed only in the H-FIRE protocol (Group 6), wherein the V/d ratio was 1200 V/cm, leading to H-FIRE ablation of regional parenchyma (Figure 3d). These results indicate that HFE treatment, using 600 V/cm and 200 bursts, causes superficial lesions limited to the electrode insertion tracks but does not produce regional ablative lesions in brain parenchyma. Only moderate heating (<1 °C) occurred as a result of energy deposition (see below). This modest temperature increase and the short treatment duration are associated with the lack of detectable tissue damage attributable to thermal necrosis.

The trend test showed a positive correlation between histological grade and time elapsed following treatment (*p* < 0.0001). A separate statistical analysis was conducted by placing groups into 4 categories; the sham group, immediate response (1–48 h and Burst_100_), delayed response (72–96 h), and the H-FIRE protocol. A Fisher exact test revealed differences among these categories (*p* < 0.0001).

### 2.4. Secondary Electrical, Thermal, and Accelerometry Measurements

#### 2.4.1. Electrical Impedance Spectroscopy for Monitoring Changes in Tissue Impedance

**Figure 4:**
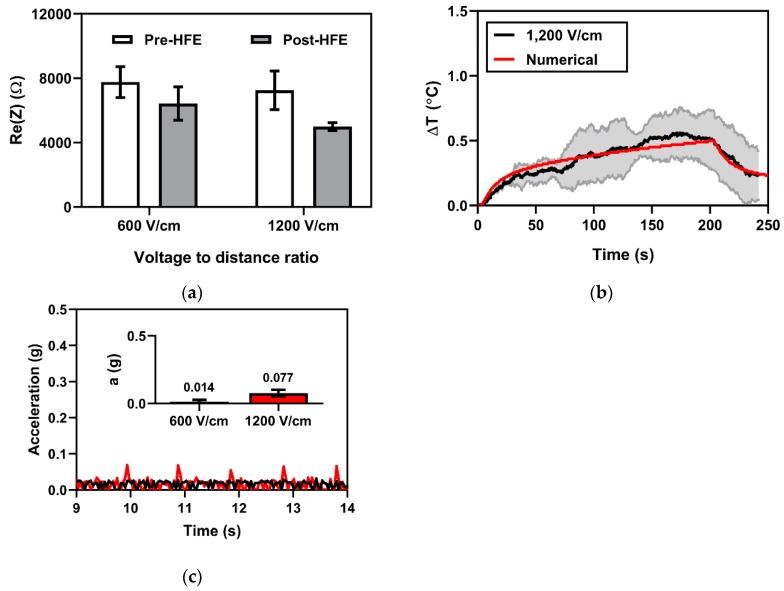
Secondary impedance, thermal, and accelerometry measurements. (**a**) Electrical impedance measurements at 20 kHz before and after HFE indicated a statistically significant change in impedance from baseline for an applied voltage-to-distance ratio of 1200 V/cm (*p* = 0.0012) and for 600 V/cm (*p* = 0.0287). (**b**) Joule heating effects immediately adjacent to the electrodes were minimal (ΔT_max_ = 0.55 °C). (**c**) Acceleration was not detected at an applied voltage-to-distance ratio of 600 V/cm but was detected at 1200 V/cm (accel = 0.077 g).

Previously, changes in tissue impedance have been proposed as an indicator of treatment outcome for electroporation-based therapies, as they provide information about ablation size and extent of permeabilization; here, we employ electrical impedance spectroscopy to measure changes in tissue impedance. Tissue electrical impedance was recorded over a frequency range of 10 kHz to 1 MHz for treatment Groups 1 (600 V/cm) and 6 (1200 V/cm). Here, we present the changes in the real part of electrical impedance prior to and after HFE treatment (Figure 4a) at 20 kHz. A baseline Re (Z) of 7764.2 ± 962.3 Ω and 7257.0 ± 1198.0 Ω was recorded for Groups 1 and 6, respectively. After HFE, tissue impedance measured 6428.4 ± 1033.6 Ω and 4993.9 ± 239.1 Ω for treatment Groups 1 and 6, respectively. A *t*-test for non-pooled variance did not demonstrate a significant difference (*p* = 0.2030) in the changes in tissue impedance before and after treatment between the 600 V/cm group (−1335.73 Ω) and the 1200 V/cm groups (−2263.16 Ω). The changes in tissue impedance in response to pulsing, for both the 600 V/cm group (*p* = 0.0287) and 1200 V/cm group (*p* = 0.0012), is significantly different from 0.

#### 2.4.2. Fiber Optic Temperature Sensing for Monitoring Joule Heating Effects

To delineate the HFE-mediated BBBD from thermal mechanisms, Joule heating effects due to HFE treatment are monitored using a fiber optic temperature sensor. Fiber optic thermal measurements taken for treatment Group 6 (1200 V/cm) showed a temperature rise below <1 °C for all treatments. The average of the temperature profiles indicated a maximum temperature rise of 0.55 ± 0.18 °C immediately adjacent to the electrode—the theoretical location for maximal temperature rise. An overlay of the experimental and numerical temperature profile (Figure 4b) demonstrated good agreement between the theoretical and observed data. A mixed effects statistical model was used to establish whether the difference between experimental temperature profile and numerical temperature profile is 0; no significant difference was detected (*p* = 0.9903).

#### 2.4.3. Accelerometry Measurements for Muscle Excitation

HFE employs bipolar pulsed EFs to mitigate muscle excitation relative to traditional monopolar pulsed EFs; therefore we utilized an accelerometer to quantify muscle contractions associated with HFE treatment. In every treatment group, muscle contractions associated with HFE were negligible. Accelerometry was unable to detect gross movement attributed to the HFE treatment for treatment groups with applied V/d ratios of 600 V/cm, though an applied V/d ratio of 1200 V/cm resulted in a measured acceleration of 0.077 ± 0.027 gs (Figure 4c). Visible muscle excitation was restricted to hemifacial contractions and contractions behind the ear. No subjects experienced cardiac arrhythmias or adverse effects due to treatment.

## 3. Discussion

Treatment of intracranial tumors and other CNS disorders (Parkinson’s disease and Alzheimer’s disease, for example) often rely on intraparenchymal drug delivery for therapeutic efficacy; this process is complicated when molecular agents are unable to permeate through the BBB and often results in ineffective treatment. The ability to produce focal and transient BBBD would provide a therapeutic window for enhanced drug delivery. Here, we demonstrate that high frequency electroporation (HFE) can open the BBB for up to 3 days post-HFE treatment. To the best of our knowledge, this is the first study to investigate the duration of HFE-mediated BBBD in vivo and to numerically quantify BTTs at various timepoints.

To achieve focal BBBD, we used two monopolar electrodes to create a non-uniform EF distribution. This allowed us to study BBBD as a result of varied local EFs (Figure 6e) with very high EFs (>1000 V/cm) immediately adjacent to the electrodes and very low EFs (<100 V/cm) millimeters away. Gd-EBD was administered systemically via IP injection 1 h prior to sacrifice; this allows sufficient time for the solution to flow and diffuse into areas of BBB permeation. It was assumed here that the distribution of capillaries and blood vessels was uniform throughout the rodent brain and the Gd-EBD solution circulated uniformly throughout the rodent brain. At an applied V/d ratio of 600 V/cm, the 1 h BBBD volume was 81.2 ± 7.9 mm^3^ based on the data generated by evaluation of fixed/stained tissue sections. Finite element analysis was subsequently implemented to map the computed EF distribution to the measured BBBD volume in tissue sections. We determined that a BTT of 113.5 ± 8.2 V/cm closely matched the BBBD volume measured at the 1 hour time-point. Interestingly, regions of tissue exposed to this local EF did not sustain BBBD for durations longer than 1 h. On the contrary, the 72 h time-point BBBD volume measured 6.4 ± 1.1 mm^3^ with a numerically determined BTT 542.5 ± 51.5 V/cm; at preceding timepoints up to the 72 h mark, tissues exposed to this EF demonstrated BBBD. Measurements at 24 and 48 h showed intermediate results, consistent with this trend. It should be noted that we consider it probable that the small size, *n*= 2, of the sham group constrained our capacity to detect a statistically significant difference from sham for both 48 h and 72 h. Previous studies have indicated no BBBD in sham groups due to electrode insertion [26,43]; therefore, we determined the number of sham rats to be *n* = 2 in order to minimize the animals sacrificed in this study. Within the context of this study, we observed that the duration of BBBD was dependent on the local EF. The corollary is that the EF distribution can be modified to produce pockets of BBBD with controlled durations of permeation; as such, a desired therapeutic window can be programmed using numerical models of the local EF distribution. In future studies, alternate electrode configurations, such as flat plate electrodes for creating a uniform EF distribution, will be used to quantitatively describe and verify the relationship between the local electric field and the duration of BBBD.

In addition to spatial characterization of BBBD, EBD was used to measure to extent of newly permeable molecular uptake by measurement of EBD fluorescence within the brain parenchyma and within blood serum. In every group, EBD fluorescence from serum was at least 1318.3 ± 61.6 µg/g, more than two orders of magnitude higher than intraparenchymal EBD fluorescence; therefore, we assume EBD uptake to the brain was not limited by circulating EBD. Tissue damage associated with electrode insertion was expected to contribute to uptake of EBD, and the sham group did show a slight (0.2 ± 0.03 µg/g) uptake of EBD into the brain. In HFE-treated groups, there was a detectable increase in the intraparenchymal EBD fluorescence at 1, 24, 48, and 72 h, but not 96 h; these data further indicate the transient nature of HFE-mediated BBBD, which, given our chosen set of pulse parameters, resolves within 96 h. Though this data does not provide information on the spatial or CNS compartmental distribution of EBD, this data is agreement with our results from MRI and gross pathology. These results are also comparable to previously observed BBBD measured 96 h after application of traditional IRE. More specifically, a large BBBD volume was observed in healthy rats within 1 hour following monophasic IRE [27]; though these volumes were not quantified, follow-up scans taken 4 days after treatment showed limited BBBD, which is comparable to our findings. The slightly longer BBBD duration resulting from traditional IRE may be attributable to differences in the amount of energy applied, as our bursts of bipolar pulses had an amplitude of 240 V, roughly four times lower than the 1000 V pulse amplitude previously used [27]. Differences in tissue response to HFE waveforms, which target intracellular components more efficiently than monophasic IRE [33], could be causing BBBD in a manner which is mechanistically different than monophasic IRE, thereby producing shorter-lived BBBD at the benefit of mitigating muscle/nerve excitation.

For clinical application of HFE-mediated BBBD, tissue ablation may not be desirable. In this study, H&E staining was used to verify that nonlethal BBBD was achieved. No tissue damage was observed proximate to the electrode insertion track up to 48 h after treatment, as indicated by histologic grade 1 scoring (Table 2 and Figure 3a). However, 72 h after HFE administration, signs of an inflammatory response, with varying degrees of meningeal infiltrates, were observed (Table 2 and Figure 3b,c). The H-FIRE protocol, 1200 V/cm, designed to ablate parenchymal tissue did indeed lead to necrosis, even far removed from the electrode insertion tracks (Table 2 and Figure 3d). Just as was previously demonstrated for traditional IRE, we have shown HFE with a V/d ratio of 600 V/cm can induce BBBD, and this is associated with minimal tissue damage. Our raising of the voltage resulted in a substantial increase in BBBD volume but also presented with remote parenchymal tissue necrosis consistent with H-FIRE-mediated cell death. These findings are consistent with prior studies that used intracranial IRE and H-FIRE; the degree of BBBD and cell death can be manipulated by proper selection of pulse parameters such as burst number and applied voltage [26,43]. Given that we observed inflammatory infiltrates within the brain parenchyma at later timepoints, it appears that HFE-induced BBBD is also associated with induction of a sterile inflammatory response similar to what has been observed with focused ultrasound-induced BBBD [44]. Theoretically, additional BBBD could be observed in association with maturation of this inflammatory response. Future studies examining the tissue effects of HFE over longer periods of time will be required to further characterize this response.

Blood–brain barrier disruption can, in the short term, also be mediated by hyperthermia [45]. To delineate potential thermal impacts on the measured BBBD in this study, a FOT sensor was adhered to the tip, at the theoretical location of the highest temperature gradient. Unfortunately, since the diameter of the FOT sensor was larger than that of the electrodes used here, these sensors imposed an additional mechanical disruption of tissue upon electrode/FOT insertion. Therefore, we chose to measure temperature for only *n* = 2 each for 1200 V/cm and 600 V/cm. Since there was a negligible temperature increase at 600 V/cm, we report the ΔT only for Group 6 (1200 V/cm). On average, the maximum ΔT was 0.55 ± 0.18 °C; this ΔT is well below temperatures known to cause thermal coagulative necrosis [46]. Since the electric field decreases exponentially away from the electrode, so too will the Joule heating from HFE. This means any temperature effects should drastically diminish away from the electrodes and result in temperature rises far below what was measured in locations with BBBD.

Accelerometry measurements at a V/d ratio of 600 V/cm did not reveal detectable movement; however, at 1200 V/cm, hemifacial contractions and detectable movement suggest higher voltages are likely to lead to muscle excitation. To counteract the effects of HFE on excitable tissues like muscle and nerves, higher frequency waveforms, or shorter pulse durations (<2 µs), could be used as opposed to 5 µs pulses [47]; as previously demonstrated, longer pulse durations typical of IRE (100 µs) are more likely to induce muscle/nerve excitation [32]. On the contrary, shorter pulse durations require a higher voltage to achieve a similar ablation in healthy tissue if the other pulsing parameters are maintained; in malignant cells, H-FIRE selectivity seems to benefit from using higher frequency waveforms [38,48,49,50]. The relationship between pulse width, cell kill, and tissue excitation has been modestly investigated [34,51], so future efforts to find the optimal pulsing parameters may prove beneficial for H-FIRE therapy. Future applications may utilize a current cage configuration to control or limit the zone of muscle/nervous stimulation, applying a precedent wherein a current cage was used to reduce the volume of tissue exposed to a 5 V/cm muscular excitatory threshold, instead of a two needle configuration [52]. We did not study the effects of HFE on nervous stimulation outside of muscle excitation, but they are interesting to consider. Specifically, vasodilation mediated by nerve stimulation [53] may have an effect on observed or experienced accumulation and clearance of Gd-EBD or therapeutic agents, respectively. Since HFE mitigates nervous stimulation, vasodilation effects may be reduced relative to traditional IRE, leading to more stable, predictable BBBD dynamics; HFE-induced nervous stimulation, vasodilation, and BBBD dynamics will be the focus of future studies.

Finally, electrical impedance measurements at 20 kHz show significant changes in tissue impedance before and after HFE/H-FIRE treatment, regardless of the applied voltage. In the H-FIRE protocol (1200 V/cm), a significant difference in tissue impedance from baseline was recorded (−2263.16 Ω), as expected. Previously, impedance changes before and after treatment have been proposed as a metric by which to assess ablation outcome [54,55]. Our histological analysis suggests tissue ablation resulting from 1200 V/cm treatment, consistent with a large decrease in our electrical impedance measurements. On the contrary, the 600 V/cm treatment (Group 1) did not demonstrate regional tissue ablation, though electrical impedance measurements were also significantly different from baseline (−1335.73 Ω). Although the mean change in tissue impedance is higher in the irreversible electroporation protocol than in the reversible electroporation protocol, the means were not statistically different from each other.

One limitation in our approach to numerical analysis is the assumption that the observed BBBD volume is topologically consistent with the computed EF contour enclosing the same volume of tissue. It is likely that CSF fluid flow pathways and other local variations in tissue electrical properties alter the boundaries of the experimental EFD. Our observations suggest that the measured boundaries of the BBBD zone will follow slight deviations from the boundaries of the isovolumetric computed field domain. This concern, associated with tissue impedance variability, was one of the factors that originally motivated the creation of H-FIRE [33]. The use of high frequency waveforms has been shown to effectively mitigate the differences in tissue impedance relative to low frequency IRE pulses [33,41]. In addition, the quantification of the sigmoidal relationship between conductivity and local EF, a relationship typically characterized from a multitude of data points, was, in our study, quantified using only two voltage conditions. While the experimental voltage and current data match well with those numerically computed, further characterization is needed to accurately quantify this complex relationship. Conductivity data reported here serves only as an approximation for rat brain tissue electrical response to HFE.

The approach we took to quantify BBBD volume relied on systemic circulation of the Gd-EBD solution for one hour prior to sacrifice. For example, at the 24 h time point, the Gd-EBD solution was administered IP 23 h after HFE; 1 hour after that IP injection, the animal was sacrificed and then either transported for MRI BBBD characterization or necropsied prior to brain tissue preservation. One limitation for this method of characterizing BBBD is that it does not quantify the dynamics of Gd clearance or recovery of the BBB. Furthermore, investigation of the variability in BBB permeability using molecules of different sizes or configurations was not included in this study. While the BTTs determined in this study describe BBBD pertaining to Gd (~950 Da) and EBD (which forms complexes with albumin measuring ~70 kDa), these thresholds may vary depending on the size and mobility of the molecule/drug in question. Some agents of interest include the chemotherapeutic anti-cancer drug methotrexate (~450 Da) and carbidopa (~225 Da), a BBB-impermeable drug used in mitigating the symptoms of Parkinson’s disease. Future studies to investigate BBB permeability to molecules of varying sizes and the clearance of these molecules are of utmost importance.

HFE demonstrates potential as a unique tool to selectively permeate the BBB. By modifying the pulsing parameters and the electrode configuration, HFE can be used to selectively open the BBB with spatiotemporal control of the therapeutic window. Ultimately, we foresee a treatment regimen wherein high-frequency pulses are used to ablate a tumor core, while the tumor margins are subjected to a combination of H-FIRE selective cell kill and HFE-BBBD-mediated exposure to precisely targeted chemical, biomolecular, or immunological agents centimeters from the solid mass [56]. HFE-induced BBBD alone is a promising development for the prospect of improved patient outcomes from those suffering from glioblastoma multiforme. Combinatorial therapy, including the selective use of HFE-mediated BBBD, would constitute a highly selective yet grossly effective therapy for brain tumor elimination. As evidenced in recent studies demonstrating a robust adaptive and innate immune response to tumors following electroporation-based therapies [57,58]; HFE now may evolve as a potential therapy not only targeting early stage tumors, but also late-stage and metastasized cancers.

## 4. Materials and Methods

### 4.1. Assurances and Surgical Procedures

The study was performed in accordance with the principles of Guide for the Care and Use of Laboratory Animals and was approved by the Institutional Animal Care and Use Committee (IACUC#16–156). Study animals were adult male Fischer rats, weighing between 170 and 215 g. Prior to surgery (craniectomy), rats were premedicated with a subcutaneous (1 mg/kg) injection of buprenorphine (Buprenoprhine SR-LAB; Zoopharm, Windsor, CO, USA), anesthetized using isoflurane induction (3%–4%:95% isoflurane:oxygen mixture), and then maintained with isoflurane (2–3.5%:95% isoflurane:oxygen mixture) delivered via nosecone. The dorsum of the head from the intercanthal area to the cranial cervical region was clipped and prepared for aseptic surgery. Anesthetized rats were instrumented in a small animal stereotactic headframe (Model 1350M; David Kopf Instruments, Tujunga, CA, USA). A unilateral rostrotentorial surgical approach to the skull was performed and a 5 mm × 2.5 mm rectangular, parietal craniectomy defect was created in the skull of each rodent using a high-speed electric drill (Dremel 3000 Series; Mount Prospect IL, USA) with a 2.4 mm diameter, round burr. Following completion of the craniectomy, two blunt-tipped stainless steel electrodes were advanced into the brain using the micromanipulator arm of the stereotactic frame according to stereotactic coordinates referenced to the location of the rostral electrode (bregma 4 mm caudal, 3.5 mm lateral, at a depth of −4 mm relative the surface of the dura). Upon placement of the electrodes, secondary electrical, thermal, and accelerometry measurements (see below) were recorded and HFE treatment commenced.

Following HFE pulse delivery and secondary measurements, the electrodes were retracted, the craniectomy defect covered with bone wax (Ethicon), and the skin incision closed with 4-0 monocryl interrupted skin sutures (Ethicon, Somerville, NJ, USA). Rats were recovered from anesthesia and monitored until their predetermined survival endpoints.

### 4.2. High-Frequency Electroporation and Parameter Selection

**Figure 5:**
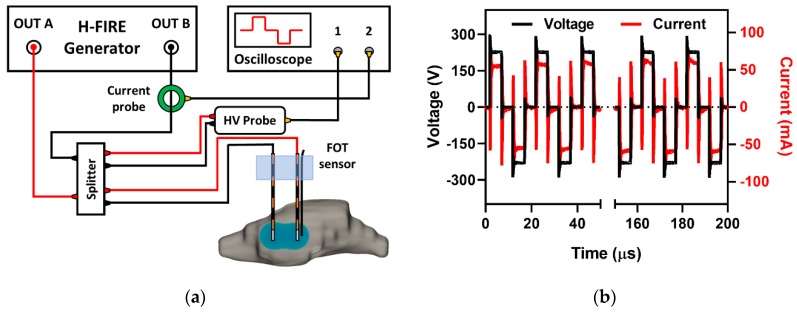
Experimental setup and voltage/current recording. HFE treatment (**a**) was delivered across two blunt tipped monopolar electrodes (ϕ = 0.45 mm, 4 mm spacing, 1 mm exposure) using a custom-built high-frequency pulse generator. The 5-5-5 µs voltage and current waveforms (**b**) were recorded using a WaveSurfer 3024 z oscilloscope with a 10 × Pearson current probe and a 1000 × high voltage probe.

A custom-built bipolar pulse generator (EPULSUS-FBM1-5, Lisbon, Portugal) [59] was used to deliver bursts of bipolar pulsed EFs to the output stainless steel electrodes (ϕ = 0.45 mm, 4 mm center-to-center spacing, 1 mm electrode exposure). This generator consisted of two unipolar Marx generators capable of producing voltage waveforms in a bipolar manner with pulse risetimes of 100 ns and a maximum voltage/current output of 5kV/50A. Voltage and current waveforms were recorded using a WaveSurfer 3024z oscilloscope (Teledyne LeCroy, Chestnut Ridge, NY) with a 1000 × high voltage probe (Enhancer 3000, BTX, Holliston, MA) and 10 × current probe (2877, Pearson Electronics, Palo Alto, CA), as seen in Figure 5a. A single burst of bipolar pulses consisted of 10 cycles, each with a 5 µs positive phase, a 5 µs intra-pulse delay, and a 5 µs negative phase (5-5-5 µs) for a total energized time of 100 µs per burst delivered at 1 Hz (Figure 5b). Unless stated otherwise, the applied V/d ratio and number of bursts were 600 V/cm and 200 bursts, respectively.

BBBD was investigated at timepoints 1, 24, 48, 72, and 96 h after HFE treatment (Table 3). Groups 1–5 comprised the temporal characterization arm of this study, in which a total of 200 bursts were applied at a V/d ratio of 600 V/cm. In addition to the temporal study, the effect of applied V/d ratio and number of bursts was investigated at the 1 hour timepoint. Group 6 was used for the investigation of BBBD due to increasing the V/d ratio from 600 (group 1) to 1200 V/cm (group 6), while maintaining other pulsing parameters; this group represents the H-FIRE protocol. Group 7 was used for the investigation of BBBD due to a decrease in number of bursts from 200 (group 1) to 100 bursts (group 7), while maintaining all other pulsing parameters. Groups 1, 5, 6, and 7 consisted of *n* = 4; groups 2, 3, and 4 consisted of *n* = 8; and the sham group consisted of *n* = 2. The sham group underwent the craniectomy surgical procedure and had the monopolar electrodes inserted into the brain, but with no pulsing administered.

### 4.3. Magnetic Resonance Imaging and Gd-EBD BBB Disruption Volumetrics

To assess BBB permeability, anesthetized rodents received an IP injection of a solution (Gd-EBD) formulated with 0.1 mmol/kg of gadopentetate dimeglumine (Gd; Magnevist; Bayer, Whippany, NJ, USA) and 75 mg/kg of 2.5% Evans blue dye (EBD; Sigma; St. Louis, MO, USA). Gd and EBD are too large to permeate the intact BBB, meaning their presence in the brain parenchyma would indicate BBBD following HFE treatment. In all cases, the Gd-EBD solution was administered 1 h prior to sacrifice; for example, at the 24 h time point, the Gd-EBD solution was administered IP 23 h after HFE. Specifically, in the 1 h timepoint, Gd-EBD was administered 5 min prior to HFE treatment and imaged 1 h post-HFE. This provided enough time for Gd-EBD to circulate systemically and permeate in regions of BBBD. Anesthetized rats were then euthanatized by IP pentobarbital (0.5 mL) overdose (Fatal Plus, Vortech Pharm, Dearborn, MI, USA) at predetermined timepoints. Brain MRI images were obtained immediately (<10 min) after euthanasia on a Philips 1.5T scanner (Intera, Philips Healthcare, Andover, MA, USA) equipped with an 8-channel head coil. T1-weighted spin echo was obtained using the following parameters: TR = 450 ms, TE = 15 ms, FOV = (40 mm)^2^, slice thickness = 2.0 mm.

Following MRI, BBBD volumes were quantified as regions of contrast enhancement in T1W scans. 3D reconstruction of BBBD regions was implemented using Osirix (Osirix MD, Bernex, Switzerland); a Gd threshold intensity was determined based on prior studies that utilized reference tubes of known Gd concentrations [26].

### 4.4. Histomorphologic Analyses of Gd-EBD Treated Rodents

Following MRI imaging, the brain of each rodent was removed. The brains of half of the rats in each treatment group were immersion-fixed in 10% neutral buffered formalin solution. Following fixation for 48 h, the brain of each rodent was placed in an adult rodent matrix slicer (Ted Pella Inc., Redding, CA, USA) and serially sectioned in the transverse plane at 2 mm intervals. Each transverse brain slice was digitally photographed (Nikon D5100, Nikon, Japan) and paraffin embedded individually in a tissue cassette. The positions of the transverse sections at which the EBD was first and last visible (the anterior/rostral and posterior/caudal limits of the z-plane of the BBB disruption) were co-registered to the corresponding channels of the brain matrix and recorded. Transverse brain sections containing EBD within these defined rostral and caudal limits were serially sub-sectioned in the transverse plane at 10 µm thickness and 200 µm intervals using a microtome and mounted on positively-charged microscope slides. Digital photomicrographs (Nikon Eclipse Ni-E, Nikon, Japan) of the intraparenchymal EBD were obtained from all intervening transverse sections using a charge-coupled device camera with a fixed aperture (Nikon DS-Fi1c, Nikon, Japan). The volume of EBD resulting from the transverse image stack from each rat was calculated using a Cavalieri estimator on a commercial image analysis system (Stereo Investigator; MBF Bioscience, Williston, VT, USA).

Brain sections within the region of EBD uptake from each rat were stained routinely with hematoxylin and eosin (H&E) and examined using light microscopy for evidence of treatment-associated brain injury. Histologic changes in the brain were reviewed independently by three investigators blinded to the treatments groups and graded using a modification (Table 4) of a previously reported system for evaluation of catheters for treatment of central nervous system disease [60]. The grading scores reported (Table 2) are the highest grade assigned for each observation.

### 4.5. Quantification of Evans Blue Dye

Brain tissue and blood samples were processed using a previously described dye extraction method [61]. Briefly, blood samples were centrifuged for 10 min at 10,000 × *g* at 4 °C. The supernatants were aspirated and mixed (1:3 v/v) with 50% trichloroacetic acid (TCA; dissolved in 0.9% saline, Sigma; St. Louis, MO, USA). This solution was then centrifuged (10,000 × *g* for 10 min at 4 °C). The resulting supernatant was collected, diluted with 50% TCA (1:300 v/v), and then again in 95% ethanol (1:3 v/v). The brain samples were homogenized in 50% TCA (1:3 w/v) using a steel-bead homogenizer (Beadblaster, Thomas Scientific, Swedesboro, NJ, USA), centrifuged (10,000 × *g* for 10 min at 4 °C), and the supernatants collected and diluted with 95% ethanol (1:3 v/v) prior to spectrophotometric determination of EBD fluorescence. The final TCA extracted supernatants were loaded onto a 96-well plate in duplicate (30 µL/well), and EBD fluorescence of blood and brain tissues was determined using a spectrophotometer (620 nm excitation/680 nm emission; SpectraMax Plus, Molecular Devices, San Jose, CA, USA).

### 4.6. Numerical Determination of BBBD Temporal Thresholds

To determine BTTs, a numerical model was constructed using COMSOL Multiphysics v5.4 (COMSOL Inc., Stockholm, Sweden). BTTs were determined numerically as the EF contour which encloses the same volume of tissue as the BBBD volume from gross pathological tissue sections. This analysis assumes the measured BBBD was topologically consistent with computed EF contour.

**Figure 6:**
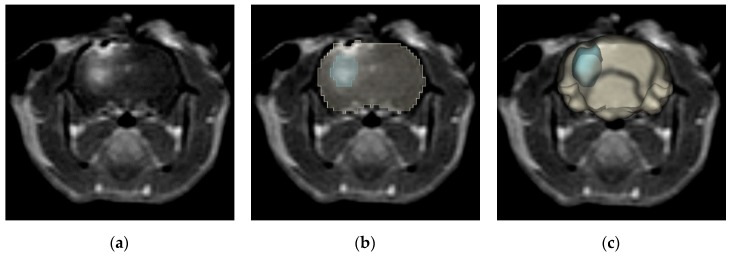

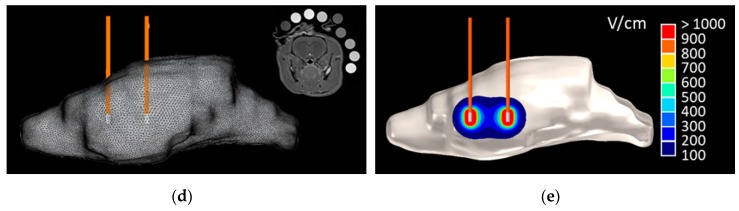
3D numerical reconstruction and BBBD temporal threshold analysis. The 3D numerical reconstruction process is as follows: from a (**a**) T1W MRI scan (Group 1), a (**b**) region selection tool was used to highlight the brain and BBBD. The brain region was (**c**) reconstructed in 3D, electrodes placed, and (**d**) meshed for numerical analysis. (**e**) The resulting electric field distribution was used to determine BBBD temporal thresholds as the EF contours which enclose the same volume of tissue as the BBBD volumes from gross pathological tissue sections.

A realistic tissue domain was defined through 3D reconstruction of a rat brain from a T1W MRI scan (Figure 6a–c) using 3D Slicer 4.10 (Slicer, https://www.slicer.org/) [62]. The final domain, including the brain and two monopolar electrodes, consisted of 298,218 tetrahedral elements resulting from an “extra fine” mesh setting within COMSOL (Figure 6d). After mesh generation, the electric potential distribution was modeled using Equation (1).

(1) ∇⋅(σ∇ϕ)=0

In Equation (1), σ represents the electrical conductivity as a function of the electric field, E, and ϕ is the electric potential. Since σ(E) for rat brain tissue has not been previously characterized, experimental voltage and current waveforms collected in this study were used to generate an approximate electrical conductivity curve of rat brain tissue using a methodology previously implemented by Sel et al. [63]; physiologic temperature was recorded as T = 32.2 °C. In total, an *n* = 30 V/I recordings from the 600 V/cm group and an *n* = 2 V/I recordings from the 1200 V/cm group were used for this analysis. A parametric study was implemented across two parameters fitted to a sigmoidal tissue response (Equation (2)). Parameters σ_0_ (0.05–0.15 S/m) and σ_f_ (0.05–0.35 S/m) were varied until the electric current predicted from the numerical model closely matched the first-burst experimental voltage and current. The remaining parameters, A (0.003) and E_del_ (1,750 V/cm), were held fixed as these parameters did not significantly alter the predicted voltage and current values from the numerical model. A sigmoidal tissue response has previously been used to represent changes in tissue conductivity due to electroporation [63]; the sigmoid in Equation (2) was used in this study.

(2) $$\sigma \left(E\right)=\;\sigma_{0}+\frac{\sigma_{f}-\sigma_{0}}{1+e^{-A\cdot \left(E-E_{del}\right)}}$$

Here, σ_0_ represents the tissue conductivity at an un-electroporated state; σ_f_ represents the electroporated tissue conductivity; A the slope of the sigmoid transition region; and E_del_ the accompanying transition range. Following the characterization of rat brain tissue conductivity, an electric potential boundary condition (ϕ = 240 V) and a grounding boundary condition was applied on either electrode. All remaining external boundaries were assumed as electrically insulating (dϕ/dn = 0). It should be noted that only two voltages were investigated in this study; therefore, the resulting sigmoidal conductivity data serves only as an approximation of the electrical tissue response to HFE.

Thermal dissipation and Joule heating effects were calculated using a modified bioheat equation (Equation (3)):

(3) $$\rho c\frac{\partial T}{\partial t}=\nabla \cdot \left(k\nabla T\right)-\omega_{b}\rho_{b}c_{b}\left(T-T_{b}\right)+\frac{\sigma \cdot \;{\left|E\right|}^{2}\cdot p}{\tau }$$

where ρ is the tissue density; c the specific heat; k the thermal conductivity; and ω_b_ the distributed blood perfusion coefficient. In this study, T_b_, ρ_b_, and c_b_ were 32.2 °C, 1050 kg/m^3^, and 3617 J/(kg∙K), respectively. The terms p and τ represent the duty cycle normalization terms, which allow for HFE thermal contributions to be represented as a continuous heat source rather than a periodic heat source, as the latter would require drastic changes in solver time-stepping. Here p is the burst on-time (100 × 10^−6^ s) and τ is the period of burst delivery (1 s). Additional parameter values used in this model are represented in Table 5.

### 4.7. Secondary Electrical, Thermal, and Accelerometry Measurements

#### 4.7.1. Electrical Impedance Spectroscopy for Monitoring Changes in Tissue Impedance

Brain tissue impedance changes were monitored using a Gamry Reference 600 potentiostat (Gamry, Warminster, PA, US). Following stereotactic placement of the blunt-tipped electrodes and prior to HFE treatment, baseline brain tissue electrical impedance was measured between 10 kHz and 1 MHz at 10 points per decade. Immediately (<5 s) after HFE treatment, a secondary impedance sweep was conducted, with the same bandwidth and resolution, in order to record changes in tissue impedance. Here, we report the changes in the real part (Re) of the complex tissue impedance at 20 kHz. This frequency was selected to reflect changes in extracellular tissue impedance.

#### 4.7.2. Fiber Optic Temperature Sensing for Monitoring Joule Heating Effects

Changes in tissue temperature, due to Joule heating effects, were monitored for the H-FIRE protocol (Group 6) and HFE protocol (Group 1) using a general-purpose STB fiber optic temperature (FOT) probe (LumaSense, Santa Clara, CA, USA). Because the diameter of the FOT probe was larger than that of the electrodes used here, these probes imposed an additional mechanical disruption of tissue upon electrode/FOT probe insertion; therefore, data collection was limited to Group 1 and 6. The FOT probe was attached along the length of a single blunt-tipped monopolar electrode and stereotactically guided into the brain tissue; temperature was recorded at a frequency of 2 Hz using a Luxtron m3300 Biomedical Lab Kit (LumaSense, Santa Clara, CA, USA). The experimental thermal data was compared to thermal changes predicted by the numerical model for further numerical validation.

#### 4.7.3. Accelerometry Measurements for Muscle Excitation

A 6-axis gyroscope/accelerometer (InvenSense MPU-6050, San Jose, California, USA), configured to an acceleration sensing range ± 4 g, was sutured to the flank of each rat at the thoracolumbar junction using 4-0 monocryl suture (Ethicon, Somerville, NJ, USA). Accelerometry was recorded at 30 Hz for groups 1, 2, and 6. Given the minimal acceleration observations from these treatment groups, no further accelerometry measurements were collected.

### 4.8. Statistical Analysis

The analysis was performed using SAS software (SAS Inc, Cary, NC). We deemed the threshold for declaration of statistical significance to be *p* < 0.05. A KW test was used to compare the pathological BBBD volumes across treatment groups. The temporal characterization arm, the V/d ratio comparison, and the burst number comparison were analyzed separately due to differences in the energy delivered in each treatment group. A KW test was also used to compare the brain and blood EBD across the treatment groups. In cases where the KW test showed significance, Dunn’s test was used for pairwise comparison between the treatment groups and the sham group. To evaluate the consistency of our two BBBD metrics, the difference between BBBD volumes measured from pathological tissue sections and from MRI (V_Path_-V_MRI_) was taken; a paired-t test was used to determine whether the difference was statistically different from 0.

Numerical validation was only conducted for two electric potentials; due to the small sample size (*n* = 2) in the H-FIRE protocol (1200 V/cm), we did not perform the goodness of fit test for this validation. The comparison was done qualitatively.

Histologic grade was assigned by 3 reviewers. The highest grade was recorded for each observation. Fisher’s exact test was used to examine the association between treatment group and grade level. For the purposes of this test, treatment groups were further grouped into four categories: the sham group, the immediate response (1–48 h and Burst_100_), the delayed response (72–96 h), and the H-FIRE protocol. We also performed the linear trend test to assess whether the histologic grade is higher with increased time following treatment.

To compare whether the pre- and post- impedance measurements are the same for group 1 (600 V/cm) and the H-FIRE protocol (group 6, 1200 V/cm), a paired-t test was used. To further compare the pre-post change between 600 V/cm and 1200 V/cm, a two-sample *t*-test was used.

Comparison between the numerical and experimental Joule heating was accomplished by taking the difference between the temperature profiles (ΔT_num_−ΔT_exp_). The mixed effects model was used to statistically evaluate whether this difference diverged from 0. Random intercept was used to account for the correlated structure.

## 5. Conclusions

Predictable, focal blood–brain barrier disruption (BBBD) was achieved using high frequency electroporation (HFE). Muscle contractions and tissue damage due to HFE treatment at a voltage to distance ratio of 600 V/cm were minimal. MR imaging and gross pathological evaluation confirmed uptake of the Gd-EBD solution at all timepoints earlier than 96 h. After 72 h there was a noticeable decrease in detection of the Gd-EBD solution, indicating transient BBBD. Numerical modeling demonstrated a high correlation between the duration of BBBD and a corresponding BBBD temporal threshold. These data indicate that HFE induces BBBD at low electric fields and that the application of higher local electric fields can be utilized for longer duration (~3 days) of BBBD.

## Figures and Tables

**Table 1:** BBBD Volumetric, EBD Fluorescence, and Numerical Results (Mean ± Standard Deviation).

Temporal (h)	Pathological BBBDV (mm^3^)	MRI BBBDV (mm^3^)	Cerebral [EBD] (µg/g)	Serum [EBD] (µg/g)	BBBD Temporal Threshold (V/cm)
sham	0.0 ± 0.0	0.0 ± 0.0	0.2 ± 0.0	1494.0 ± 0.0	∞
1	81.2 ± 7.9 *	84.1 ± 8.7 *	18.5 ± 0.29	1318.3 ± 66.8	113.5 ± 8.2
24	47.1 ± 15.1 *	40.9 ± 5.4	10.9 ± 0.45	1393.8 ± 122.0	174.9 ± 37.1
48	9.9 ± 1.1	10.4 ± 1.1	4.0 ± 0.31	1404.0 ± 115.1	432.7 ± 30.8
72	6.4 ± 1.1	5.8 ± 1.0	1.2 ± 0.13	1403.3 ± 145.1	542.5 ± 51.5
96	0.0 ± 0.0	0.0 ± 0.0	0.3 ± 0.04	1400.5 ± 107.5	∞

* denotes a *p*-value < 0.05 for the comparison with the sham group of each column, respectively.

**Table 2:** Histologic grade assignment by treatment group.

Category	Survival (h)	Grade 1	Grade 2	Grade 2a	Grade 2b	Grade 3a	Grade 4
sham	sham, *n* = 2	2	0	0	0	0	0
Immediate response	Burst_100_, *n* = 2	2	0	0	0	0	0
	1, *n* = 2	2	0	0	0	0	0
	24, *n* = 4	4	0	0	0	0	0
	48, *n* = 4	4	0	0	0	0	0
Delayed response	72, *n* = 4	1	1	2	0	0	0
	96, *n* = 4	0	0	2	2	0	0
H-FIRE	H-FIRE, *n* = 2	0	0	0	0	1	1

A Fisher exact test revealed differences among the categories (*p* < 0.0001). The trend test showed a positive correlation between histological grade and time elapsed following treatment (*p* < 0.0001). “n” represents the number of rats examined using light microscopy with H&E staining.

**Table 3:** Experimental matrix; HFE-mediated BBBBD was investigated 1–96 h post-HFE.

Group	Time-Point (h)	Voltage/Distance Ratio (V/cm)	Number of Bursts
sham **^§^**	1	0	0
1 *	1	600	200
2 **^†^**	24	600	200
3 **^†^**	48	600	200
4 **^†^**	72	600	200
5 *	96	600	200
6, H-FIRE *	1	1200	200
7, Burst_100_ *	1	600	100

**^†^** represents (*n* = 8); * represents (*n* = 4); **^§^** represents (*n* = 2).

**Table 4:** Modified histological grading scheme [60].

Score	Criteria: Brain Parenchyma Changes
0	No lesions apparent
1	Superficial cerebral contusion, edema, electrode tracks, +/- hemorrhage; lesions limited to electrode tracks
2	Superficial cerebral contusion, edema, electrode tracks, +/- hemorrhage, inflammation; lesions limited to electrode tracks
3	Parenchymal hemorrhagic necrosis/ablation localized to electrode tracks/tips
4	Regional parenchymal hemorrhagic necrosis/ablation (necrosis around and remote from needle tracks)
	Criteria: Meningeal Changes *
a	Mild meningeal inflammatory infiltrates; limited locally to electrode insertion areas
b	Moderate meningeal inflammatory infiltrates; meningeal involvement throughout surgical site
c	Severe meningeal inflammatory infiltrates; diffuse meningitis extending beyond surgical field

* could accompany any Grade 0–4 lesion.

**Table 5:** Electrical and thermal values for the numerical model.

Material	Parameter	Value	Units
Brain tissue	Density, ρ	1046	kg/m^3^
	Specific heat, c	3630	J/(kg∙K)
	Thermal conductivity, k	0.51	W/(m∙K)
	Blood perfusion coefficient, ω	1.75 × 10^−3^	1/s
Insulation	Density, ρ	1190	kg/m^3^
	Specific heat, c	1470	J/(kg∙K)
	Thermal conductivity, k	0.18	W/(m∙K)
	Electrical conductivity, σ	2.5 × 10^14^	S/m
Stainless steel	Density, ρ	7850	kg/m^3^
	Specific heat, c	475	J/(kg∙K)
	Thermal conductivity, k	44.5	W/(m∙K)
	Electrical conductivity, σ	4.0 × 10^14^	S/m

Material properties were attained from the IT’IS database (https://itis.swiss).
